# Changes in Astrocyte Shape Induced by Sublytic Concentrations of the Cholesterol-Dependent Cytolysin Pneumolysin Still Require Pore-Forming Capacity

**DOI:** 10.3390/toxins3010043

**Published:** 2011-01-07

**Authors:** Christina Förtsch, Sabrina Hupp, Jiangtao Ma, Timothy J. Mitchell, Elke Maier, Roland Benz, Asparouh I. Iliev

**Affiliations:** 1 DFG Membrane, Cytoskeleton Interaction Group, Institute of Pharmacology and Toxicology &amp; Rudolf Virchow Center for Experimental Medicine, University of Würzburg, Versbacherstr. 9, 97078 Würzburg, Germany; Email: foertsch@toxi.uni-wuerzburg.de (C.F.); shupp@toxi.uni-wuerzburg.de (S.H.); 2 Division of Infection and Immunity, Level 2, Glasgow Biomedical Research Centre, University of Glasgow, 120 University Place, Glasgow, G12 8TA, UK; Email: j.ma@bio.gla.ac.uk (J.M.); t.mitchell@bio.gla.ac.uk (T.J.M.); 3 Rudolf Virchow Center for Experimental Medicine, University of Würzburg, Versbacherstr. 9, 97078 Würzburg, Germany; Email: emaier@biozentrum.uni-wuerzburg.de (E.M.); roland.benz@mail.uni-wuerzburg.de (R.B.)

**Keywords:** pneumolysin, pore formation, cytoskeleton

## Abstract

*Streptococcus pneumoniae *is a common pathogen that causes various infections, such as sepsis and meningitis. A major pathogenic factor of *S. pneumoniae* is the cholesterol-dependent cytolysin, pneumolysin. It produces cell lysis at high concentrations and apoptosis at lower concentrations. We have shown that sublytic amounts of pneumolysin induce small GTPase-dependent actin cytoskeleton reorganization and microtubule stabilization in human neuroblastoma cells that are manifested by cell retraction and changes in cell shape. In this study, we utilized a live imaging approach to analyze the role of pneumolysin’s pore-forming capacity in the actin-dependent cell shape changes in primary astrocytes. After the initial challenge with the wild-type toxin, a permeabilized cell population was rapidly established within 20-40 minutes. After the initial rapid permeabilization, the size of the permeabilized population remained unchanged and reached a plateau. Thus, we analyzed the non-permeabilized (non-lytic) population, which demonstrated retraction and shape changes that were inhibited by actin depolymerization. Despite the non-lytic nature of pneumolysin treatment, the toxin’s lytic capacity remained critical for the initiation of cell shape changes. The non-lytic pneumolysin mutants W433F-pneumolysin and delta6-pneumolysin, which bind the cell membrane with affinities similar to that of the wild-type toxin, were not able to induce shape changes. The initiation of cell shape changes and cell retraction by the wild-type toxin were independent of calcium and sodium influx and membrane depolarization, which are known to occur following cellular challenge and suggested to result from the ion channel-like properties of the pneumolysin pores. Excluding the major pore-related phenomena as the initiation mechanism of cell shape changes, the existence of a more complex relationship between the pore-forming capacity of pneumolysin and the actin cytoskeleton reorganization is suggested.

## 1. Introduction

*Streptococcus pneumoniae* (pneumococcus) is a human pathogen that causes life-threatening diseases, such as pneumonia, sepsis, and the most common form of bacterial meningitis. Disease rates are particularly high in young children, elderly people and immunosuppressed patients [1]. New strains that are resistant even against potent reserve antibiotics continuously occur. Not only developing but also highly developed countries are confronted with the threat of *S. pneumoniae* infection [2]. Bacterial meningitis is associated with high lethality and neurological disability in the surviving patient. Only 30% of the infected patients overcome the disease, and 30% of these survivors are affected by long term sequelae [3], including mental retardation, learning impairment and focal neurological deficits (e.g., hearing loss). The acute infection is accompanied by blood vessel inflammation, CNS necrosis, neuronal loss and general inflammation of brain tissue [4]. 

Proper functioning of adult neurons in the brain is known to be crucial for learning and memory [5], and it is believed that alterations in learning and memory are a common consequence of toxic factor exposure [6]. Neuronal function and brain homeostasis in turn are strongly dependent on functional glial cells, such as astrocytes, oligodendrocytes and microglia. Whereas microglia are small, highly–motile macrophage-like brain components, astrocytes are big star-shaped, process-bearing cells, that constitute up to 50% of the volume of most brain areas, and outnumber neurons by over fivefold [7]. Connected to each other via gap junctions, and thereby forming a glial syncytium, they fence in neurons and oligodendrocytes. Astrocyte end-feet are also a major component of the blood–brain barrier together with an endothelial cell layer that controls the exchange of substances between blood and brain [8,9]. Astrocytes play a critical role in synapse mediator uptake and recycling in the brain as well [10]. Dysfunction of astrocytes and microglia contributes to dysfunction of neurons and *vice versa*. Thus, not only neuronal alterations, but also damage to or disturbed functioning of glial cells should be considered to occur during the course of bacterial meningitis [11].

*S. pneumoniae* carries at least eight specific proteins, which determine its pathogenicity. A major virulence factor is the protein toxin pneumolysin (PLY), which is produced by virtually all clinical isolates [12]. PLY is a cytosolic protein that is predominantly released upon bacterial lysis [13], although other mechanisms are also suggested [14]. It belongs to the family of cholesterol-dependent cytolysins (CDC). The hallmark of these cytolysins is their binding to the cell membrane in a cholesterol-dependent manner and penetration through the host’s membrane by producing a pore. The structural information concerning PLY has been determined by fitting the structure of perfringolysin O (PFO) to PLY [15]. PLY consists of four domains, arranged in an asymmetric manner. The currently accepted pore formation model describes PLY monomers binding to membrane cholesterol with their C-terminal domain 4 via a Trp-rich motif to form a prepore. PLY penetrates the membrane following the unfolding of the molecule and by inserting domain 3 into the lipid environment of the membrane [15,16]. Thus, a barrel-structured pore with a size of ~260 Å is formed, which leads to membrane collapse and cell lysis. 

Most of the structural biology studies use PLY concentrations of 10 µg/mL to 200 µg/mL and test its effects on artificial liposomes, artificial membranes or erythrocytes [16,17,18]. In pathological conditions, such as *S. pneumoniae* meningitis, the concentration on PLY in the cerebrospinal fluid (CSF) of patients never exceeds 0.2 µg/mL [19]. In an effort to study PLY effects in the context of the immune system, non-pore forming mutants were developed. Two of them-delta6-PLY and W433F–PLY—were used in this work. These variants are incapable of lytic pore formation at pathologically relevant concentrations. At high concentrations, W433F-PLY shows 1% hemolytic activity when compared to the WT-PLY, but should be capable of forming ion channel-like pores at concentrations of 1-10 µg/mL in planar lipid bilayers (PLB) [16]. Delta6-PLY is incapable of forming pores, as the mutation is located between domain 1 and 3, thus losing the capability to oligomerize [17,20]. Here, two amino acids are deleted, A146 and R147 [17]. It remains, however, unclear whether lytic pore formation and micropore (possessing ion channel-like properties) formation, both of which are properties of CDCs, are mechanistically similar or different phenomena.

Many bacterial toxins have developed an evolutional ability to either directly or indirectly (by altering the modifying biochemical cascades) reorganize cellular actin [21]. One final effect of these changes is a change in the cellular motility and displacement [22], which leads to improved bacterial entrance through tissue barriers. We have shown that PLY leads to the activation of the small GTPases RhoA and Rac1 in the SH-SY5Y human neuroblastoma cell line [22]. In rodent meningitis models, PLY-deficient strains exhibit a reduced virulence, characterized by strongly impaired brain invasion and CSF distribution [23,24]. Obviously, PLY plays an important role in the early infection development [25] and demonstrates immunomodulatory effects [26]. Unfortunately, large amounts of the cell biology features of PLY are studied in cell lines, but not in primary cells [22,27], which makes translation of results to primary cell systems complicated. Furthermore, it remains unclear whether these effects are seen in a similar manner in primary brain cells and whether pore formation is required for these changes or not. To clarify the role of pore formation in the reorganization of the actin cytoskeleton, as well as to translate the finding to primary brain cells, we investigated the effects of WT-PLY and the two non-lytic mutants delta6-PLY and W433F-PLY in primary astrocytes.

## 2. Materials and Methods

### 2.1. Pneumolysin Preparation

W433F-PLY [16] (partially non-lytic (1% of wild-type) mutant) is expressed in *Escherichia coli* XL-1 cells (Stratagene, Cambridge, UK) and purified by hydrophobic interaction chromatography as described previously [17,28]. The non-toxic delta6 version of the plasmid was constructed by site–directed mutagenesis (QuikchangeSDMKit, Stratagene) of pET33bPLY [29] using primers 23B [30] and 23C [30] to introduce a deletion of alanine at position 146 and arginine at position 147. WT-PLY and delta6-PLY were then expressed in *E. coli* BL21 (DE3) cells (Stratagene) and purified using nickel affinity chromatography and anion exchange chromatography, as previously described [30]. All purified proteins were tested for the presence of contaminating Gram-negative LPS using the colorimetric LAL assay (KQCL-BioWhittaker, Lonza, Basel, Switzerland). All purified proteins had <0.6 Endotoxin Units (EU)/μg protein. The primers were as follows:

23B-GGTCAATAATGTCCCAATGCAGTATGAAAAAATAACGGCTC

23C-GAGCCGTTATTTTTTCATACTGCATTGGGACATTATTGACC

### 2.2. Cell Cultures, Vital Staining, Live Imaging

Primary astrocytes were prepared from the brains of postnatal day 4-6 newborn Sprague-Dawley (SD) rats or C57BL/6 mice and cultured as a mixed culture as previously reported [11] in DMEM medium (GibcoBRL, Invitrogen GmbH, Karlsruhe, Germany), supplemented with 10% fetal calf serum (FCS) (PAN Biotech GmbH, Aidenbach, Germany) and 1% penicillin/streptomycin (GibcoBRL) in 75 cm^2^ poly-L-ornithine (Sigma-Aldrich Chemie GmbH, Schnelldorf, Germany) coated cell culture flasks (Sarstedt AG & Co., Nuembrecht, Germany). At day 10-14 after seeding, the astrocytes were trypsinized and seeded in chamber slides, coated with poly-L-ornithine. 

For live imaging experiments, the cells were incubated in pH-insensitive L-15 Leibovitz medium (Invitrogen) at 37 °C with propidium iodide or YOYO-1 green chromatin stain in the medium, thus staining permeabilized cells, and Hoechst 33342, staining the nuclei of all cells (all stains diluted 1:1,000 of a 1 mg/mL stock, Invitrogen). The cells were visualized on an Olympus Cell^M Imaging system using 10× and 20× dry objectives (Olympus Deutschland GmbH, Hamburg, Germany). Membrane potential was visualized by cell incubation with the potential-sensing fluorescent dye DiBac4(5) (bis-(1,3-dibutylbarbituric acid) pentamethine oxonol; 500 nM, Invitrogen) using an Olympus Cell^M system or a Leica LSM SP5 microscope (Leica Microsystems Heidelberg GmbH, Mannheim, Germany). The signal background was measured for 5 min before further treatments. 

Astrocyte transduction with GFP tagged to the membrane localization fragment, was performed using a baculovirus vector system (Organelle Lights expression system, Invitrogen) according to manufacturer’s instructions.

Confocal imaging was performed using a heated chamber system at ~37 °C, acquiring images every 2 minutes (DiBAC4(5)) at an image resolution of 1024 × 1024 pixel.

In all experiments, cells and tissues were treated with PLY in serum-free medium. In live imaging experiments, the cells were starved for 60 min-12 h before exposure to the toxin. Cell displacement and retraction was analyzed by simultaneous observation of toxin-treated and mock-treated cells with automatic repositioning of the microscopic stage with a precision of 1 µm (according to manufacturer’s instructions) per move. The cells were tracked by ImageJ (version 1.43 for Windows, National Institute of Health, Bethesda, MD, USA), using a stack tacking plug-in from the microscopy plug-in package of Tony Collins (http://rsbweb.nih.gov/ij/plugins/mbf-collection.html). 

### 2.3. Special Buffers and Treatments

Membrane hyperpolarization of astrocytes was achieved by exposure to 200 mM sucrose (Carl Roth, Karlsruhe, Germany) [31]. The effect of sucrose in our experimental system was titrated by continuous monitoring with DiBac4(5) until stable and reproducible hyperpolarization was achieved at 200 mM sucrose. In calcium (Ca)-free buffer experiments, the cells were exposed to the toxin, in a buffer containing 135 mM NaCl, 2.5 mM MgCl_2_, 4mM KCl, 5 mM Hepes (all from Carl Roth), pH = 7.3. Sodium-free buffer contained 135 mM tetramethylamonium chloride (TMA; from Carl Roth), 2.5 mM MgCl_2_, 4 mM KCl, 5 mM Hepes, pH = 7.3. Thapsigargin (3 µM, Enzo Life Sciences GmbH, Lörrach, Germany) was applied for 30 min in Ca-free buffer, to empty the intracellular depots [32] before further treatment was performed. In cholesterol inactivation experiments, PLY was treated with cholesterol (Sigma) in a ratio of 1:10 (toxin:cholesterol, w/w) in PBS for 30 min at 37 °C, as the cholesterol solvent (ethanol, Carl Roth) never exceeded 0.1% in the inactivation solution. In some experiments, the treatment medium was collected after 30-60 min challenge of the cells, PLY was inactivated by cholesterol and the medium was transferred to a new test culture in order to study the role of products, released after cell lysis. 

### 2.4. Immunohisto- and Immunocytochemistry

The cells were fixed with 4% paraformaldehyde in PBS (all from Sigma). They were permeabilized with 0.1% Triton X-100 (Carl Roth) for 10 min at room temperature (RT), blocked with 4% BSA in PBS for 30 min RT and incubated with primary antibody against vinculin (Santa Cruz) or with phalloidin-Alexa488 (Invitrogen) for 1 h RT. Goat anti-mouse Fab fragment, labeled with FITC or Cy3 (1:200; Dianova, Hamburg, Germany) were used to visualize the primary antibody. In some experiments, the nuclei were stained with Hoechst chromatin stain. All samples were preserved with Mowiol (Carl Roth, Germany). Samples were visualized with a Leica LSM SP5 microscope, using a 63× oil immersion objective. Image processing and analysis was performed by ImageJ. For focal adhesions analysis, equivalent imaging areas in the middle of each culture well (4-well chamber slides, Nunc, Thermo Electron LED GmbH, Langenselbold, Germany) with equivalent cell density were visualized, as microglia cells (isolectin B4-positive (isolectin B4 from Sigma)) were excluded and only astrocytes (as judged by positive anti-GFAP (Sigma) immunostaining) were analyzed. All measurements were performed on a slice at the bottom of the cells after obtaining a complete confocal z-stack of each imaging field.

### 2.5. Protein Biochemistry Analysis

In spectrophotometric experiments, tryptophan fluorescence of recombinant PLY (0.5 µM) was analyzed in a buffer comprising 50 mM Tris acetate (pH = 7.2) and 100 mM potassium acetate. The fluorescence spectrum between 300 and 400 nm following excitation at 280 nm was analyzed using a Perkin Elmer LS50B (Perkin Elmer, Inc., Waltham, MA, USA) spectrophotometer, before and after the addition of cholesterol (or ethanol (both from Sigma) as a solvent control) in a ratio of 1:1 (w:w) cholesterol:PLY. 

In Western blot experiments, samples containing equal amounts of proteins were electrophoresed on SDS-PAGE gradient gels (4-12% acrylamide, Anamed, Germany). The separated proteins were transferred via semi-dry blotting to PVDF Imobilion-P transfer membrane (Millipore, Germany). Membranes were blocked using 3-5% non-fat milk (Carl Roth, Germany) and were incubated with anti-PLY antibody (Abcam Inc., Cambridge, MA USA) (1:400). After incubation with a horseradish peroxidase-conjugated rabbit anti-mouse antibody (Jackson Immuno Research, USA) blots were detected using ECL Plus Western Blotting Reagent (GE Healthcare, Germany) and a the Fluorchem Q machine (Alpha Innotech, Germany). 

Primary rat astrocytes were mechanically homogenized by passing them through a G19 needle in 200 µL lysis buffer (containing 250 mM sucrose and a protease inhibitor mix (Roche Applied Science, Mannheim, Germany). The homogenate was centrifuged at 900 g for 10 min at 4 °C. The resulting supernatant was centrifuged at 110,000 g for 75 min at 4 °C. The crude membrane pellet was resuspended in lysis buffer. 

### 2.6. Lactate Dehydrogenase (LDH) Test

The lactate dehydrogenase detection kit (Roche Diagnostics GmbH, Mannheim, Germany) was used in some experiments, according to the manufacturer’s instructions to assess toxicity and cell lysis.

### 2.7. Planar Lipid Bilayer (PLB)

The planar lipid bilayer experiments were carried out as previously described [33]. Membranes were formed from a 1% (w/v) solution of diphytanoyl phosphatidylcholine (PC) (Avanti Polar Lipids, Alabaster, AL, USA) in n-decane. 25% cholesterol (w/w) was added to simulate cholesterol content in an astrocyte membrane. The toxin (0.1 µg/mL-0.3 µg/mL) was added to the aqueous phase after the membrane had turned black. The membrane current was measured with a pair of Ag/AgCl electrodes with salt bridges switched in series by a voltage source and a highly sensitive current amplifier (Keithley 427, Keithley Electronics, Garland, TX, USA). The temperature was maintained at 20 °C throughout.

### 2.8. Evaluation and Statistics

Image processing and image analysis were performed using ImageJ software (version 1.43 for Windows, National Institute of Health, Bethesda, MD, USA). Statistical analysis was performed on GraphPad Prism 4.02 for Windows (GraphPad Software Inc., La Jolla, CA, USA). Statistical tests included a Mann-Whitney U-test (comparing 2 groups differing with one parameter at a time) or one–way ANOVA with Bonferroni post-test (comparing 3 or more groups, differing with one parameter at a time).

## 3. Results and Discussion

**Figure 1:**
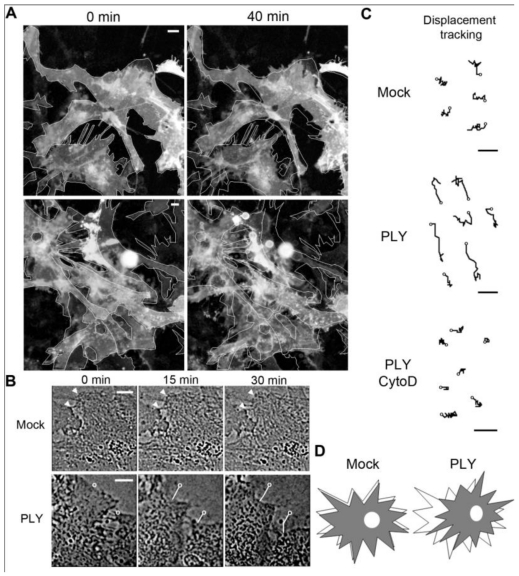
Effects of pneumolysin on primary astrocytes. (**A**) Mouse astrocytes, transduced with baculovirus, encoding membrane-targeted GFP, respond with cell shape changes and retraction to 0.1 µg/mL PLY. Cell contour outlines indicate time point 0, allowing better comparison. (**B**) Example of cell retraction and cell border tracking following PLY challenge. Scale bars: 10 µm. (**C**) Increased cell displacement by 0.1 µg/mL PLY. Actin depolymerization by 4 µM cytochalasin D (CytoD) blocked the PLY-induced changes. Circles indicate the tracking starting points. Scale bars: 5 µm. (**D**) Schematic presentation of the displacement-shrinking and deformation, rather than enhanced migration.

Previously, we have demonstrated small GTPase-dependent actin remodeling in SH-SY5Y neuroblastoma cells by pneumolysin at sublytic concentrations [22]. When exposing primary rodent glial cells (originating from SD rats or C57Bl/6 mice) to 0.1 µg/mL PLY, cell retraction and displacement were observed (Figure 1(A), baculovirus-based membrane-GFP live imaging labeling of astrocytes). The astrocytic nature of the cells was further verified by an anti-GFAP immunostaining (not shown). Detailed analysis demonstrated retraction of individual cells that were adherent to the surface, which was accompanied by retraction of processes [Figures 1(A,B)]. In larger astrocyte clusters, directional displacement was observed (probably due to accumulation of cell shape changes; see Supplementary data S1 and S2 for movies). These effects occurred within 30 minutes after toxin challenge. Software tracing of the cell edges (Figure 1(B) shows a tracing example) allowed for visualization and measurement of the exact magnitude of cell retraction and displacement. Actin depolymerization by cytochalasin D fully inhibited the displacement of the astrocytes after PLY exposure [Figure 1(C)]. 

**Figure 2:**
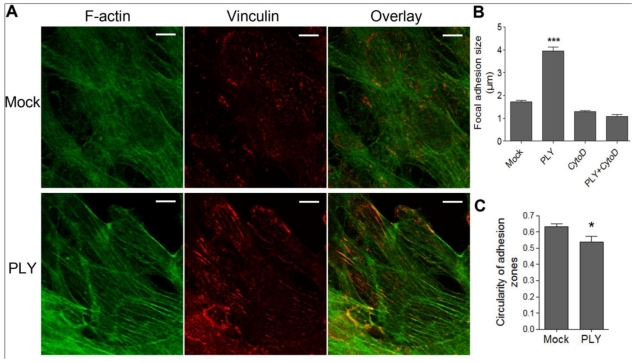
Induction of focal adhesions following PLY challenge. (**A**) Immunostaining of monolayers of primary astrocytes with phalloidin for F-actin (green) and vinculin antibody for focal points/adhesions (red). (**B**) Increase of focal adhesions size 30 min after challenge with 0.1 µg/mL PLY, which is fully inhibited by actin depolymerization following 4 µM cytochalasin D (CytoD) exposure. *** p < 0.001 *vs.* all. Values represent mean ± SEM, n = 20-40 cells. (**C**) Focal adhesion shape change from circular (closer to 1) to elongated (closer to 0) following PLY challenge, indicative for a transition from focal points to focal adhesions. * p < 0.05, Values represent mean ± SEM, n = 40-60 cells, >5,000 focal points analyzed. Scale bar: 10 µm.

Since active cellular retraction and displacement in a given direction should be accompanied by changes in the focal adhesion formation and size [34] due to the establishment of traction points, we labeled focal adhesions by vinculin immunocytochemistry [35]. Before PLY challenge, the astrocytes demonstrated defined, but small focal points, which increased to focal adhesions within 30 min after toxin exposure [Figures 2(A,B) (size of adhesions), C (shape of adhesions-more circular (focal points] or elongated (focal adhesions)), indicative of RhoA GTPase-like activation and analogous to our earlier experimental findings in neuroblastoma cells [22]. The increase in focal adhesion size was also inhibited by cytochalasin D pretreatment [Figure 2(B)].

These studies helped us to further verify that the cell shape changes represent active cytoskeleton reorganizing phenomena without a loss of surface attachment [36]. A model describes the relationship between adhesion and motility, which recognizes three states-weak, intermediate and strong adhesion state [37] (for a further review see [38]). The presence of focal points in the astrocytes in normal, untreated conditions indicated a capacity to be motile. The cell displacement and retraction effects, followed by a less motile phase, accompanied by increased adhesion as witnessed by the progression from focal points to focal adhesions, fit very well with the concept of a transition from intermediate to strong adhesion separated by a motile step.

All these observations confirm that: (i) Active cytoskeleton (most probably actin) reorganizing events follow PLY challenge in primary astroglial cells; and (ii) cell displacement and membrane retraction live imaging studies can be used as a reliable and immediately accessible marker for the occurrence of actin changes following PLY treatment. This finding is not surprising, because it confirms the general knowledge that mammalian cellular motility depends on actin reorganization phenomena, as determined by numerous laboratories in the last few decades and excellently reviewed by Pollard and Borisy [39], Ridley *et al*. [40] and Pollard and Cooper [41]. The aim of the current work was to only investigate the role of the pore-forming capacity of PLY as an initiation factor for the actin cytoskeletal reorganization phenomena, rather than studying in a detail the molecular cascades that link PLY and the actin changes. The reason is that toxin-induced actin-reorganizing phenomena in glial cells seem to be much more complex than those observed in human neuroblastoma cells, and some non-small GTPase-dependent events might contribute to their occurrence (manuscript in preparation). Cell displacement and retraction, in contrast, is a cumulative effect, which can be used as a suitable qualitative marker for the capacity of the toxin or its mutant form to initiate cytoskeleton reorganizing phenomena. 

**Figure 3:**
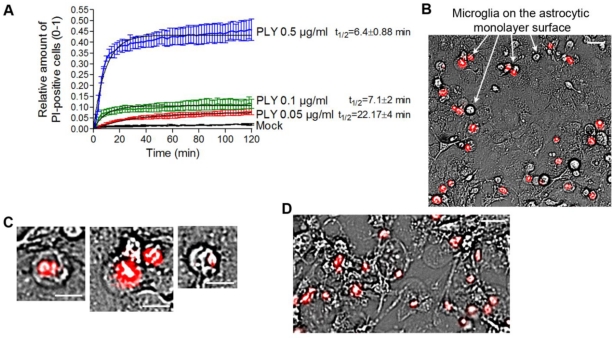
Analysis of the permeabilized/non-permeabilized cells. (**A**) Permeabilization curves of primary mouse astrocytes following challenges with different concentrations of PLY as judged by PI chromatin staining. Values represent mean ± SEM, n = 3 experiments. (**B**) Microglia (arrows) represent nearly all of the permeabilized cells in a primary glial culture. Scale bar: 20 µm. (**C**) Permeabilized cells are round and immobile, with pyknotic nucleus. Scale bar: 10 µm. (**D**) Cell death and rounding in astrocytes following a challenge with lytic 0.5 µg/mL. Scale bar: 20 µm.

In our experiments, we always observed the presence of dose-dependent cell lysis. Since PLY is a cytolysin, we decided to precisely define the terms lytic/sublytic/non-lytic concentrations in our system in order to be able to interpret our findings correctly. We performed live imaging analysis of primary glial cultures in the presence of propidium iodide (PI), using PLY concentrations in the range of 0.05 and 0.5 µg/mL. Simultaneous staining with PI allowed for exclusion of all permeabilized cells. PI staining is suitable for discriminating acutely lytic from non-lytic cells [42], but also cells with “classical” PLY membrane pores (with a size of ~260 Å) should be easily PI-permeable (MW 650) [22]. Nevertheless, we have no firm evidence that PI could penetrate sufficiently fast through individual macropores to stain the chromatin. Furthermore, the possibility of transient macropore formation could not be excluded (and we doubt whether such transient pores might suffice to mediate PI penetration into the cytosol), therefore, we limited our definitions to lytic (permeabilized) and non-lytic (non–permeabilized) cell populations. The permeabilization of the cells occurred in an exponential manner and reached a plateau at all concentrations no later than 40 min after PLY challenge [Figure 3(A)]. As the concentration of PLY rose, the half-time of cell permeabilization dropped from ~22 min (0.05 µg/mL) to ~6 min (0.5 µg/mL). As soon as the plateau was reached, two defined populations of cells, permeabilized (PI-positive; accounting for about 10% of cells at 0.1 µg/mL PLY) and non-permeabilized (~90% of the cells at 0.1 µg/mL PLY), were formed and remained unchanged with the time. In all further experiments, we analyzed only the effects of PLY on the non-permeabilized cells. Most permeabilized cells belonged to the microglial type, and were clearly distinguishable on the top of the astrocyte monolayer in mixed cultures [Figure 3(B), arrows]. All permeabilized cells demonstrated a lack of motility, a rounded morphology of the cell body, and a pyknotic rounded nucleus shape [Figure 3(C)]. Since the baculovirus transduction works only with astrocytes (but not microglia) and most of the lysed cells were microglia, virtually all GFP-positive cells in Figure 1 should be considered intact. When challenging cells with 0.5 µg/mL PLY, however, many of the astrocytes in the monolayer were also permeabilized and demonstrated a rounded cellular morphology without processes [Figure 3(D)], and were clearly different from the motile astrocytes under the influence of the toxin. We also excluded the existence of transient lytic pores by using a sequential staining of the nuclei with PI and YOYO-1 (a green chromatin stain), and showed that cells, once permeabilized, remained permeable without a reversal of the permeabilization status (not shown). We have also analyzed the possibility that the lytic changes in some of the cells release bioactive components that induced cell shape changes in the rest of the astrocytes. For this purpose, we treated glial cells with high lytic amounts of PLY, collected the medium, inactivated the toxin by short exposure to cholesterol [22] and challenged fresh new glial cell preparations with this medium under live imaging conditions. None of the cells that were exposed to the lytic products of the lysed glial cells demonstrated retraction, increased motility or changes in cell shape, as seen in cells treated with pneumolysin (not shown). Thus, one could speculate that in non-lytic conditions, PLY induces partial displacement and retraction, followed by an increase of focal adhesion formation. 

**Figure 4:**
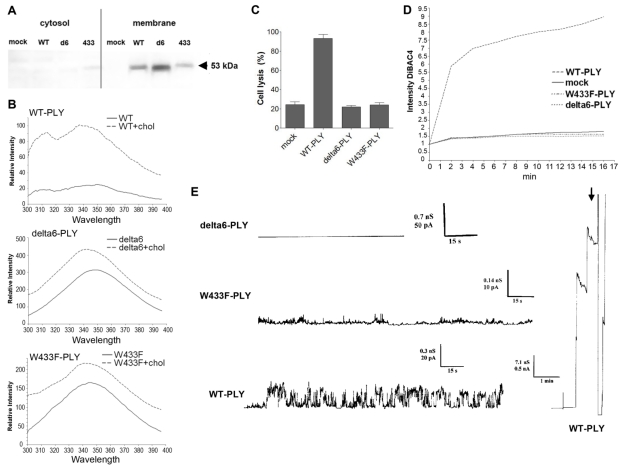
Membrane binding and pore formation capacity of PLY variants. (**A**) Western blot analysis of the membrane binding of the PLY variant at similar protein concentration. The binding difference was adjusted in all further experiments to achieve equivalent membrane toxin load (WT-WT-PLY, d6-delta6-PLY, W433F-W433F-PLY). (**B**) Spectrophotometric analysis of the tryptophan fluorescence of the PLY variants before and after incubation with cholesterol (chol), demonstrating membrane binding. (**C**) LDH release after exposure of primary astrocytes to 0.5 µg/mL WT-PLY and equivalent concentrations of the toxin variants for 30 min. Values represent mean ± SEM (n = 3 experiments). (**D**) Membrane polarity change (as judged by the fluorescence intensity of DiBAC4(5)) following exposure to 0.1 µg/mL toxin. The graph is representative of 5 experiments. (**E**) Analysis of the electric conductance in planar lipid bilayer membranes after challenge with 0.3 µg/mL WT-PLY and equivalent concentrations of the toxin variants at a voltage of 70 mV. An arrow demonstrates the strong staircase-like increase of the conductance following the formation of big pores in the WT-PLY experiment, missing in the mutants.

Whereas the role of lytic pores in actin-dependent cell displacement effects could be excluded, it remained unclear whether pore formation is needed at all. Evidence exists that PLY builds a heterogeneous population of pores, many of which have ion channel properties [43]. All these experiments, however, utilize PLY concentrations in a range exceeding the pathophysiologically relevant concentrations of PLY (up to 0.2 µg/mL in meningitis [19]) by a factor of 10 or more. Thus, we approached this obstacle by characterizing in detail the behavior of PLY at concentrations up to 0.2 µg/mL, which were entirely within the disease-relevant range, and by utilizing some non-lytic PLY mutant forms, W433F-PLY and delta6-PLY [16,17]. The mutants are known to be strongly deficient or completely incapable in their pore forming capacity: W433F-PLY shows 1% of wild-type lytic capabilities and delta6-PLY is completely non-lytic (see Introduction). Both mutants bound cell membranes in a manner similar to WT-PLY [Figure 4(A)], which was further confirmed by biochemical spectrophotometric cholesterol-binding experiments [Figure 4(B)]. The binding capacity however, was not completely equal among the pneumolysin forms, therefore the concentration of the mutants was adjusted in all further experiments to fit exactly the WT-PLY binding. As expected, none of the mutants was lytic in the range of 0.1 to 0.5 µg/mL [Figure 4(C)]. Earlier experiments in artificial membranes and cell membrane patches show that W433F-PLY form big (presumably lytic) pores at a concentration of 5 µg/mL and more [43,44]. However, these concentrations exceeded those that we used in our experiments. 

In primary glial cell cultures treated with non-lytic concentrations of PLY, the toxin induced membrane depolarization [Figure 4(D)]. Membrane polarity was analyzed using the oxonol dye DiBac4(5). This dye does not cause mitochondrial uncoupling and shows a linear fluorescence *versus* membrane penetration relationship [45]. The dye’s fluorescence increases when interacting with intracellular proteins and preferably with hydrophobic binding sites. Since PLY pores are known to be predominantly cation conductive [16,43,44], the occurrence of membrane depolarization implies a role of sodium and/or Ca influx. We cannot exclude the possibility, however, that membrane depolarization is not due to direct effects of the PLY pores but to the involvement of endogenous ion channels as previously suggested [22].

Consistent with the depolarization experiments, we tested the conductance across the membrane after challenge with WT-PLY, W433F-PLY and delta6-PLY in a PLB as described before [33]. Only the WT-PLY was capable of forming big pores (corresponding most likely to lytic pores) [Figure 4(E), arrow]. Smaller oscillations (up to 25 pA at 170 mV) were also observed, which indicated an activity on the artificial membrane. Small conductance oscillations (not bigger than 5 pA at 170 mV) were seen with the W433F-PLY mutant, although much smaller and infrequent than with WT-PLY at an equivalent toxin concentration [Figure 4(D)]. The treatment with the delta6-PLY mutant did not cause any conductance change across the membrane, *i.e.*, no pore-forming capacity was present. We could not witness the presence of micropores by W433F-PLY, as defined before [43], which could be explained by the much lower concentrations of the toxin we used and the presence of cholesterol in the PLB. 

**Figure 5:**
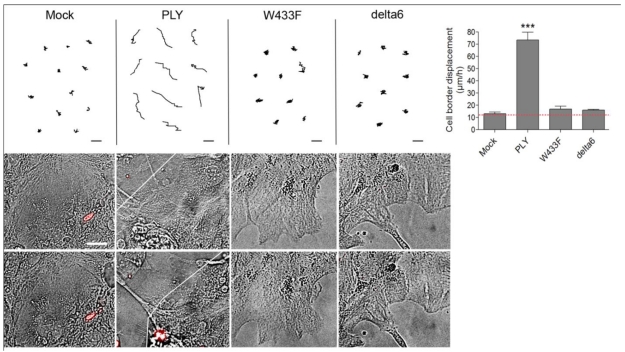
Cell displacement and retraction following exposure to PLY variants. Primary astrocytes demonstrated retraction and displacement only after exposure to 0.1 µg/mL WT-PLY, but not when challenged with an equivalent amount of the mutant variants (WT-WT-PLY, d6-delta6-PLY, W433F-W433F-PLY). Cells are incubated in the presence of propidium iodide, which detects permeabilization in few cells, but not in the retracted ones. *** −p < 0.001 *vs.* all other groups. Values represent mean ± SEM, n = 40-60 cells of 4 experiments. The red line in the graph indicates automatic stage repositioning shift. Scale bars: 10 µm.

Following the characterization of the WT-PLY and the mutants, we tested the ability of the non–pore–forming mutants to cause cell shape changes in astrocytes. Both W433F-PLY and delta6-PLY mutants failed to cause any shape change or retraction in primary astrocytes, even at concentrations exceeding the WT-PLY concentration by up to five-fold (Figure 5). Thus, although the effects of PLY affected a non-lytic population of cells, the lytic pore formation capacity remained critically important for inducing cytoskeletal cell shape changes. The possible mechanistic explanation could be limited to the formation of transient macropores, micropores (non-lytic pores with ion-channel-like features) or to non-pore-related molecular interactions that require the molecular conformation capacity of PLY. 

**Figure 6:**
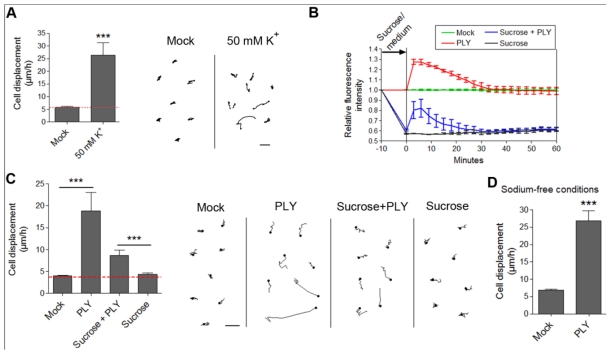
Effects of membrane depolarization on the PLY-induced cell retraction and displacement. (**A**) Membrane depolarization by 50 mM potassium leads to increased cell retraction within 30 min. *** p < 0.001. Values represent mean ± SEM, n = 20-30 cells of 3 experiments. (**B**) Changes in membrane depolarization (as assessed by the DiBac4(5) fluorescent intensity change) following 0.1 µg/mL PLY challenge in primary astrocytes with and without pretreatment with 200 mM sucrose as a hyperpolarizing agent. Values represent mean ± SEM, n = 3 experiments. (**C**) Cell retraction and displacement effects following 0.1 µg/mL PLY exposure, with and without membrane hyperpolarization by sucrose, remain. *** p < 0.001. Values represent mean ± SEM, n = 30-40 cells of 3 experiments. Scale bars: 5 µm. The red line in the graphs indicates automatic stage repositioning shift. (**D**) Cell displacement following 0.1-0.2 µg/mL PLY exposure in sodium-free conditions (TMAC replacement) remains. *** p < 0.001. Values represent mean ± SEM, n = 60-80 cells of 4 experiments.

The evidence for membrane depolarization required studying its possible role as a causative link between toxin pores and cytoskeletal changes. Membrane depolarization is capable of activating small GTPases in eye epithelial cells [46] and modulating the actin cytoskeleton via a Rho/ROCK signaling cascade in airway smooth muscles [47]. The actin-binding protein profilin I increases its colocalization with actin in neurons following membrane depolarization [48]. The actin-associated alpha-fodrin, capable of mediating actin reorganization, changes its submembranous distribution after membrane depolarization in secretory cells [49]. Membrane depolarization could be experimentally achieved by an excessive potassium (K) exposure. We incubated primary astrocytes with 50 mM K to cause rapid membrane depolarization [Figure 6(A)]. We observed retraction/displacement of the cells following K exposure [Figure 6(A)]. Analysis of GFP-stained cells (Supplementary data S4) also demonstrated the occurrence not only of cell retraction, but also of generally increased membrane/cell border dynamics compared with mock-treated cells (Supplementary data S3). Although membrane depolarization *per se* could obviously alter cellular kinetics, we needed to provide direct evidence of whether membrane depolarization could initiate the actin-dependent cell shape changes by PLY in astrocytes. For this purpose, we exposed glial cells to 200 mM sucrose to induce membrane hyperpolarization [31]. We verified this by DiBAC4(5) live imaging. Indeed, five minutes after exposure to 200 mM sucrose, the membrane polarity increased [Figure 6(B)]. We incubated the hyperpolarized cells with 0.1 µg/mL PLY and observed again toxin-induced cell displacement and retraction, not present in the control sucrose-conditioned cells [Figure 6(C)]. Notably, the motility difference (as shown of the graph) between sucrose-free and sucrose-treated cultures in the presence of PLY is not indicative of a difference in response, rather a difference in cell density because the data stem from different cultures. Essential in these tests is the comparison with the corresponding controls, which are prepared and visualized simultaneously with the PLY-treated cultures (see Methods). Although hyperpolarized, the sucrose-pretreated astrocytes demonstrated a certain level of depolarization (not exceeding the background polarity of the cells before sucrose application) following PLY challenge, which followed exactly the same depolarization curve in amplitude and shape as the PLY-challenged cells without sucrose. This result suggests that, in the presence of sucrose, membrane pore formation by the toxin remains relatively unchanged. In our experimental system, we mimicked membrane depolarization by high K; however, in a real experimental environment membrane depolarization is normally induced by sodium (as well as small amounts of Ca) influx. Potassium-selective pores would allow K-efflux and hyperpolarization under toxin influence, which is not the case in the glial experimental system, thus excluding high K selectivity. Thus, although our membrane hyperpolarization experiments confirmed that membrane depolarization alone was not an initiator of cell displacement and retraction, we could not exclude that it might play a modulatory role in the cytoskeletal reorganization effects. This could be of special importance in the context of the central nervous system, where bioelectrical phenomena have profound effects on the crosstalk glia/neurons [50]. Next, we replaced sodium chloride in the imaging buffer with equimolar tetramethylammonium chloride (thus preserving the osmolarity), eliminating sodium as an influx factor. Again, PLY was able to induce clear displacement and cell shape changes in the astrocytes despite the lack of extracellular sodium [Figure 6(D)]. 

Since ion influx via ion-selective pores should be accompanied by water influx too, we exposed glial cells to hypotonic medium after 50% reconstitution with distilled water. None of the cells demonstrated any change in shape, motility or the occurrence of cell displacement, which excluded the role of water influx and swelling alone as a possible cell shape change inductor (not shown).

**Figure 7:**
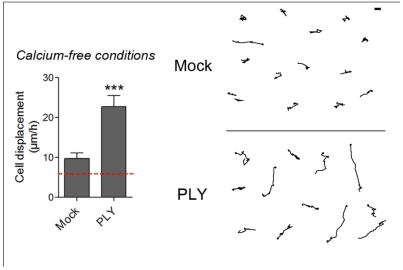
Cell displacement and retraction in Ca-free conditions. 0.1 µg/mL PLY produces cell retraction and displacement in Ca-free buffer conditions and intracellular Ca depot depletion by 3 µM thapsigargin. *** p < 0.001. Values represent mean ± SEM, n = 30-40 cells of 3 experiments. The red line in the graph indicates automatic stage repositioning shift. Scale bar: 5 µm.

The ion selectivity of PLY micropores, derived from experiments with PLBs, indicates relative cation selectivity and sensitivity against closure by bivalent ions such as Ca and zinc (“small” pores). PLY alone is known to produce an increase in cellular Ca [27], thus Ca-selective micropores should be additionally considered, assuming that they exist and play a role in plasma Ca elevation. Local increase of cytosolic Ca is a factor, contributing to cellular motility in electrical field stimulation model systems, which partially resembles the effects of PLY on membrane electrical phenomena [51,52]. The major source of Ca influx in cells after PLY challenge is the extracellular environment [27], although Ca influx from intracellular stores, the biggest of which is the endoplasmic reticulum (ER) [53], should be also considered. We have shown that in the human SH-SY5Y neuroblastoma cell line, the activation of Rac1 (but not of RhoA) by PLY is dependent on Ca influx [22]. Such an experimental setup, however, excludes the role of intracellular Ca depots, thus making it difficult to judge the role of Ca influx in the initiation of cell shape changes. To answer the question about the initiation role of Ca influx in PLY-induced cell shape changes, we tested the role of PLY on astrocytes in Ca-free medium after an additional depletion of the intracellular Ca stores by 3 µM thapsigargin (an inhibitor of the ER Ca^2+^ ATPase), as described by Ubl and Reiser [54]. Such a combination allows a smooth, but long-lasting cell Ca depletion without reducing the normal cytosolic Ca level (e.g., as done by BAPTA and other Ca chelators), thus maintaining it at a concentration needed for normal functioning of the cytoskeleton and associated myosins and just blocking its provisional increase [32]. Following PLY exposure, strong cellular displacement was initiated (Figure 7), thus providing evidence that Ca influx either from extra- or intracellular depots alone is not responsible for the initiation of these cellular changes. The mock-treated astrocytes in Ca-free buffer conditions, however, also demonstrated slightly increased motility (Figure 7), which is consistent with the evidence for modulation of the function and structure of extracellular adhesion molecules, such as E-cadherin, by Ca [55]. 

In summary, our work provides evidence for the critical importance of the pore formation capacity of PLY in cell shape changes, cell retraction and displacement in non-lytic conditions. Although we did not find evidence to support the mechanistic role of the membrane depolarization and the Ca influx as pore-related cell shape change initiation factors, we could not exclude their modulatory role in the cellular motility effects following the initial PLY challenge. The binding to membrane cholesterol, with or without the formation of toxin clusters, however, did not suffice for producing cell shape changes when tested with the non-lytic mutants. We cannot exclude the possibility that other pore formation-related factors play a role in the actin cytoskeleton effects of PLY. One possibility is the interaction with plasmalemmal molecules or with molecules positioned immediately under the membrane, the interactions of which would require the toxin molecules to be in a folded pore-like conformational state, thus explaining the need for pore-forming capacity. Further work is needed to clarify these issues. 

The possible explanation of the pathogenic role of these cell shape changes goes beyond the scope of this paper. Nevertheless, one could speculate that cell retraction and displacement, followed by increased adhesion, interferes with the important role of the astrocytes in efficiently isolating the brain tissue from the pathogens as a component of the blood-brain barrier. 

Although many bacterial toxins possess the feature of modulating actin cytoskeleton dynamics, mostly via small GTPase activation or inhibition [21], hardly any of these toxins possesses pore forming capacity. Our finding of small GTPase activation via PLY in human neuroblastoma cells [22] suggested a possible role not only of pore formation but also of cholesterol sequestration and others. Here we provide evidence that the pore forming capacity remains essential for rapid cytoskeletal reorganization, implying that PLY pore formation might play a much more complex role in the modulation of pathogen virulence than previously expected.

## 4. Conclusions

The cholesterol-dependent cytolysin pneumolysin, a major pathogenic factor of S. pneumoniae, produced massive cell shape changes and cell retraction in primary astrocytes. These changes occurred in non-lytic conditions, but nevertheless required the pore-forming capacity of PLY. The effects were largely independent of cellular ion flux phenomena and implied the existence of a more complex toxin/membrane/cytoskeleton interaction mechanism, in which an intact molecular pore-forming capacity remains essential without being necessarily related to pore formation.
